# Underwriter Discourse, IPO Profit Distribution, and Audit Quality: An Entropy Study from the Perspective of an Underwriter–Auditor Network

**DOI:** 10.3390/e26050393

**Published:** 2024-04-30

**Authors:** Songling Yang, Yafei Tai, Yu Cao, Yunzhu Chen, Qiuyue Zhang

**Affiliations:** 1 School of Economics and Management, Beijing University of Technology, Beijing 100124, Chinataiyf@emails.bjut.edu.cn (Y.T.); 2 School of Public Policy and Management, Tsinghua University, Beijing 100084, China; chenyunz19@mail.tsinghua.edu.cn

**Keywords:** information entropy, complex networks, underwriters, profit distribution, audit quality

## Abstract

Underwriters play a pivotal role in the IPO process. Information entropy, a tool for measuring the uncertainty and complexity of information, has been widely applied to various issues in complex networks. Information entropy can quantify the uncertainty and complexity of nodes in the network, providing a unique analytical perspective and methodological support for this study. This paper employs a bipartite network analysis method to construct the relationship network between underwriters and accounting firms, using the centrality of underwriters in the network as a measure of their influence to explore the impact of underwriters’ influence on the distribution of interests and audit outcomes. The findings indicate that a more pronounced influence of underwriters significantly increases the ratio of underwriting fees to audit fees. Higher influence often accompanies an increase in abnormal underwriting fees. Further research reveals that companies underwritten by more influential underwriters experience a decline in audit quality. Finally, the study reveals that a well-structured audit committee governance and the rationalization of market sentiments can mitigate the negative impacts of underwriters’ influence. The innovation of this paper is that it enriches the content related to underwriters by constructing the relationship network between underwriters and accounting firms for the first time using a bipartite network through the lens of information entropy. This conclusion provides new directions for thinking about the motives and possibilities behind financial institutions’ cooperation, offering insights for market regulation and policy formulation.

## 1. Introduction

Networks enhance organizational performance through knowledge transfer and information sharing (Zhao et al., 2023; Chahine et al., 2019) [1,2]. The role of underwriter networks in the IPO (Initial Public Offering) process has increasingly attracted scholarly and industry-wide attention (Rumokoy et al., 2019; Bajo et al., 2016; Chuluun, 2015) [3,4,5]. The network of underwriters connected by the lead underwriter (through participation in various IPO syndicates) plays a critical role in information extraction and dissemination during the IPO underwriting process (Corwin and Schultz, 2005) [6]. The central position of underwriters within their peer networks affects multiple IPO characteristics of the listed companies, including the likelihood and magnitude of IPO offer revisions, as well as short-term and long-term returns (Vithanage et al., 2016) [7]. Moreover, as underwriters often collaborate in syndicates during the IPO process, they naturally leverage their peer networks to enhance underwriting performance. Although underwriters can significantly impact various aspects of the IPO through access to additional information and channels via their relationship networks, research on underwriter relationship networks remains insufficiently explored, especially regarding their connections with other financial institutions. This paper aims to better examine the role of underwriter relationship networks.

In fact, beyond the underwriter-to-underwriter relationship networks, underwriters also establish close connections with accounting firms through repeated collaborations. These connections enable underwriters to acquire valuable information more accurately and comprehensively, reducing information asymmetry between underwriters, listed companies, and accounting firms. On the other hand, close relationships between underwriters and accounting firms could potentially affect the audit independence of the accounting firms (DeFond et al., 1999) [8]. Current research has not addressed the impact of the relationship networks between underwriters and auditors on IPOs. Our study focuses on how the central position of underwriters in their relationship networks with auditors impacts the distribution of interests between the two parties.

Claude Shannon, the father of information theory, drew from thermodynamics concepts to propose in 1948 that all information contains redundancy, and the average amount of information after excluding redundancy is termed “information entropy”. As a physical quantity measuring the disorder of a system, entropy is widely used in the social sciences to analyze complex networks and uncertainty issues (Xi and Cui, 2023; Omar and Plapper, 2020) [9,10]. In underwriter relationship networks, “information entropy” quantifies the network’s uncertainty and complexity. This paper considers underwriter influence as a crucial factor affecting the flow of information and the distribution of interests during the IPO process. Specifically, the level of underwriter influence directly impacts the efficiency of information acquisition, processing, and dissemination during the IPO, thereby significantly affecting the distribution of interests. When underwriters possess high levels of influence, they can better manage and control the flow of information during the IPO, thereby influencing the ratio of underwriting fees to audit fees.

According to the theory of interest predation, nodes in a central position within a network typically possess crucial resources and power, leading to rent-seeking behaviors (El-Khatib et al., 2015; Ishii and Xuan, 2014; Fracassi and Tate, 2012) [11,12,13]. IPO underwriting is a relationship-intensive business (Carter and Manaster, 1990) [14], and China is a typical relational network society (Yin et al., 2022) [15]. Underwriters utilize their discretionary power to allocate undervalued IPOs to investors who provide them with private benefits (Zhang, 2020) [16]. In the IPO market, underwriters significantly influence the choice of auditors, effectively achieving their intentions (Pittman and Fortin, 2004) [17]. The auditors’ selection rights and the relative bargaining power of underwriters allow us to infer the resulting impacts. This inference is valuable because the opaque operations within relationship networks are inherently difficult to observe and identify.

Contrary to existing research that constructs a unimodal network from the connections formed through mutual cooperation among underwriters (the underwriter-to-underwriter relationship network), our study innovatively applies a bipartite network analysis from SNA (social network analysis) to construct the relationship network between underwriters and accounting firms. This approach characterizes each underwriter’s influence within the peer network developed through such interactions, using SNA metrics. We dynamically use each collaboration between underwriters and accounting firms over the past three years to establish connections, thereby constructing the current year’s network centrality indicators for underwriters. Underwriter influence is measured using degree centrality, closeness centrality, and betweenness centrality from SNA, along with the average of these three metrics. Section 6.1 will detail and discuss these four metrics for measuring the main underwriter’s influence, illustrated using real-world underwriter-accounting firm networks.

The empirical results of this paper can be summarized as follows: Firstly, higher influence often leads to an increase in abnormal underwriting fees, reinforcing the possibility that underwriters leverage their influence to occupy a larger share in the IPO interest distribution. Secondly, companies underwritten by those with higher influence exhibit a higher probability of restating their financial statements in the year of listing, along with less robust financial reports. From the perspective of information entropy, this phenomenon can be understood as the elevation of underwriter influence leading to an imbalance in the distribution of information during the IPO process. This paper conducted a series of robustness tests and addressed endogeneity issues, with all results remaining robust. These findings support the interest predation hypothesis within social relationship theory, indicating that underwriters leverage their position and advantages to secure more private benefits, which may lead to threats to the audit independence of the accounting firms. Lastly, further tests on how to mitigate the negative impacts of relational capital suggest that well-structured audit committee governance and rational market sentiments could alleviate the effects of underwriters’ influence to some extent. These two solutions correspond to the “information equilibrium” and “information transparency” concepts within information entropy theory, aiding in achieving a balance and transparency of information among all parties during the IPO process.

This research contributes in multiple ways. Firstly, despite the recognized importance of underwriter relationship networks, no literature has attempted to explore the impact of networks between underwriters and accounting firms. To our knowledge, this study is the first to examine such networks, where underwriters opt to collaborate by forming syndicates, a practice that significantly impacts the relationships among underwriters. Underwriters in more central positions within the network affect the IPO characteristics and underwriting performance of the companies they underwrite. It is noteworthy that the central position of underwriters within their network merely serves as an indicator of the extent of underwriter-to-underwriter contacts. Our study extends the research on underwriter relationship networks by using a bipartite network to quantify the relationships between underwriters and accounting firms.

Secondly, our research also supplements the literature on auditor selection. The choice of auditors during the IPO process is driven by multiple factors, including the company’s financial performance, litigation risk, and auditors’ perceived risk (Ghosh and Tang, 2015; Bedard et al., 2003) [18,19]. The selection of high-quality auditors is also considered a signal revealing the real value of IPO companies (Datta et al., 2024) [20]. Our perspective, focusing on underwriters rather than issuing companies (clients), aligns more closely with the realities of Chinese IPOs. In practice, companies rarely establish formal networks with accounting firms before listing. In the Chinese IPO process, obtaining an unqualified audit opinion report is crucial for issuing companies to meet regulatory requirements, making the choice of accounting firms relatively homogeneous for companies. On the other hand, underwriters lead the entire IPO process. In 2005, the China Securities Regulatory Commission (CSRC) adopted a pure book-building approach, granting underwriters greater discretion, thereby allowing them to influence the choice of auditors and achieve their ultimate objectives. Thus, in China, underwriters play a key but often overlooked role in the selection of auditors.

Thirdly, from the perspective of relational network impacts, our study has for the first time uncovered the influence of underwriters’ sway within relational networks on the distribution of benefits and audit quality between parties. Our investigation revealed that the strength of voice within the network could lead to shifts in the distribution patterns of benefits, providing new theoretical support for the strategic interplay between institutions. Additionally, our research contributes to the understanding of factors affecting audit quality. While a body of literature has examined the impact of social relationships between executive managers and auditors on audit outcomes (Guan et al., 2016) [21], studies on the effects of relationships between underwriters and accounting firms are less common. Our investigation fills this gap, demonstrating the negative impact of such relationships on the independence of accounting firms.

Beyond academic value, our study holds practical significance for regulatory bodies. Our findings indicate that underwriters with greater influence might threaten the audit independence of the accounting firms and impact audit quality. This conclusion offers new directions for thinking about the motives and possibilities behind financial institution collaborations, providing insights for market regulation and policy formulation. Recognizing the existence of internal relational networks within financial institutions and addressing the potential conflict between self-interest and independence of auditing bodies are essential considerations in the design of IPO systems.

## 2. Literature Review 

### 2.1. Underwriter Networks

The business of IPO underwriting is relationship-intensive. Thus, for underwriters, establishing and maintaining relationships is particularly important. To acquire relevant information and advance the IPO process, underwriters rely on various relationships, such as those with companies, investors, and other underwriters. Scholars have pointed out the significance of the relationship between underwriters and their clients. Additionally, the importance of networks among underwriters is increasingly emphasized. Underwriters choose to collaborate by forming syndicates. This recurrent mode of collaboration profoundly impacts the relationships among underwriters (Adams and Kastrinaki, 2022) [22]. As core intermediary institutions in the IPO process, the primary task of underwriters is to guide and coordinate various financial institutions in integrating and disclosing information, as well as to facilitate the IPO process (Busaba et al., 2001) [23]. Through the underwriter network, the lead underwriter can indirectly utilize the client and investor networks of other underwriters, thus accessing additional information and distribution channels. A high network centrality and rich industry experience afford the lead underwriter greater opportunities to influence the likelihood and extent of IPO pricing revisions (Chen et al.,2022) [24]. Similarly, lead underwriters with high network centrality tend to attract more institutional investors to hold their stocks and obtain higher analyst coverage, long-term stock liquidity, and stock investment returns, thereby bringing the IPO price closer to its intrinsic value (Lan et al., 2021) [25]. Further empirical studies have validated the close association between underwriter network centrality and underwriting performance, institutional investor participation, and the company’s long-term performance (Rumokoy et al., 2019) [3].

### 2.2. Underwriter and Accounting Firm Contact

Firstly, if underwriters choose to collaborate with well-known, reputable accounting firms, investors are more likely to trust the financial information and the reasonableness of IPO pricing of the listed companies. This can enhance investors’ trust in the listed companies and increase the market’s acceptance of the IPO (Zhao et al., 2022) [26]. Secondly, the IPO process involves numerous regulatory requirements and legal provisions. The cooperative relationship between underwriters and accounting firms ensures that companies comply with financial reporting and disclosure requirements, adhering to regulatory norms. This helps reduce potential legal risks and improve the market reputation of the listed companies. Thirdly, underwriters and accounting firms usually possess extensive professional knowledge and experience, collaborating to formulate IPO strategies and marketing plans for companies. Such cooperative relationships can effectively increase the success rate of companies during the IPO process and ensure optimal market performance (Du et al., 2018) [27].

Social network scholars quantify power from a relational perspective, offering various quantitative indicators of power (El-Khatib et al., 2021) [28]. Existing literature on underwriter networks primarily utilizes centrality measures widely used in SNA literature to assess the relative position of lead IPO underwriters in investment banking networks (Alperovych et al., 2022) [29]. However, these measures are generally based on networks constructed among underwriters within the same group. Unlike existing literature, we use a bipartite network, which extends beyond the relationship networks between underwriters, to include a second layer of networks between underwriters and accounting firms. The centrality in this dual-layer network model measures underwriters’ influence within peer networks. Three different types of centrality measures widely used in SNA literature assess the influence of lead IPO underwriters in their networks with both groups. The first measure is degree centrality, representing the number of accounting firms a lead IPO underwriter has connections with in the three years preceding the IPO year. A high degree centrality for a node underwriter indicates that it occupies a central position in the network between underwriters and accounting firms, holding the greatest power. The second indicator is betweenness centrality; an underwriter with high betweenness centrality lies on many of the shortest paths connecting accounting firms, measuring the underwriter’s degree of control over resources within the networks. The third measure is closeness centrality; the closer an underwriter is to an accounting firm, the easier it is for information to be transmitted between them. Moreover, higher closeness centrality for an underwriter also indicates less dependence on any particular accounting firm (Chaudhry et al., 2022) [30].

### 2.3. Audit Quality

Any discussion of audit quality proxies must address the difficulty of defining audit quality. (i) DeAngelo (1981) defines audit quality as the joint probability that an auditor “detects irregularities in a client’s accounting system and reports the irregularities”, and (ii) Patterson et al. (2019), who define higher audit quality as “greater assurance of high financial reporting quality”. Survey evidence from Rajgopal et al. (2021) suggests that individual investors perceive auditor competence as an indicator of high audit quality, while audit professionals perceive compliance with auditing standards as an indicator of high audit quality. Thus, DeAngelo (1981) seems to focus on auditors’ input into error detection, while practitioners focus on compliance (Rajgopal et al., 2021). The definition of Patterson et al. (2019) can be said to encompass both auditors’ detection of errors and compliance with auditing standards (Rajgopal et al., 2021) [31,32,33].

A large number of accounting studies have investigated the determinants and consequences of audit quality. Common proxies for audit quality can be categorized into output-based and input-based proxies (Syam et al., 2021) [34]. Output-based measures typically include (1) material restatements, preferably initiated by the auditor and SEC AAER; (2) going-concern opinions; (3) financial reporting characteristics, such as the use of gross accruals or the tendency of firms to meet or exceed quarterly analysts’ consensus estimates of earnings; and (4) perception-based metrics, such as earnings response coefficients, stock price reactions to auditor-related events, and cost of capital metrics (Amin et al., 2021) [35]. 

The existing literature has overlooked the significant impact that the intricate network of underwriters’ partnerships with accounting firms holds on the Initial Public Offering (IPO) process. In reality, the collaborative relationship between underwriters and accounting firms holds immense importance in determining the success and market performance of IPOs (Falconieri et al., 2019) [36]. This cooperation facilitates the transmission of vital market signals, ensures compliance with regulatory frameworks, and provides invaluable professional guidance and strategic advice (Garg, 2020) [37]. Consequently, this paper aims to widen the research horizon by exploring this often-neglected dimension of the IPO process.

## 3. Institutional Background

The Chinese market presents a variety of institutional features and mechanisms not found in other markets (Yang et al., 2022) [38], supporting our theoretical assumptions and providing a suitable setting for examining our research objectives, summarized into four points.

First, the evolution of China’s stock issuance system significantly differs from the registration-based stock market in the United States. In its initial stage (1992–2000), China adopted an administrative approval system, where the government set stock issuance quotas and made administrative recommendations, effectively a fully governmentalized measure. From 2001 to 2022, the approval system was used. Although the approval system shifted from government selection of enterprises to nurturing, selecting, and recommending market intermediaries, regulatory bodies played a substantive role in review and regulation. The highly regulated nature of China’s capital market has led to an opaque and uncertain IPO qualification application process, making IPO qualification a scarce resource in China’s capital market. Given that all parties involved in the IPO process stand to gain substantial benefits once a company successfully lists, there is a keen competition to share in the spoils. Among the issuance and listing expenses, intermediary costs constitute the largest proportion, with the fees for the sponsoring institution (lead underwriter) being the most prominent. Unlike the generally fixed percentage of total funds raised for underwriting fees abroad (Iannotta and Navone, 2008) [39], the range of underwriting fees in China is quite broad (Appendix A Table A1 presents an overview of the issuance expenses for newly listed companies from 2011 to 2022).

Notably, at the legal level, there are currently no explicit regulations, national standards, or specific industry norms to constrain intermediary fees. Article 20 of the “Securities Issuance and Listing Sponsorship Business Management Measures” (2020) only stipulates that “fees related to performing sponsorship duties shall be determined through negotiation according to industry norms”, without specific standards issued by the China Securities Association. Thus, the standard for underwriter fees largely depends on the underwriter’s bargaining power.

Second, the phenomenon of relationship economics is widely accepted in China (Chen et al., 2017) [40]. Viewed as a typical relational society, China’s market characteristics remain information asymmetry and uncertainty about corporate prospects (Megginson et al., 2014) [41]. In this context, the importance of network relationships in the Chinese market is increasingly highlighted, as they facilitate knowledge and information sharing, thereby helping to mitigate problems of information asymmetry and uncertainty. Information can be categorized into private and public. On one hand, private information flows within relationships, reducing information asymmetry among related parties. On the other hand, institutions with strong relationships often leverage these networks to seek economic benefits. Notably, the business of IPO underwriting is relationship-intensive, making it theoretically and practically significant to study how underwriters leverage relational networks to gain economic benefits in the specific context of China.

Third, there are challenges to the full independence of auditors. According to the “Chinese Certified Public Accountants Professional Ethics Code”, accounting firms should not adopt charging arrangements that result in severe adverse effects on their own interests due to direct or indirect forms of compensation, as no safeguards could reduce this to an acceptable level. However, data published on the China Securities Regulatory Commission’s official website indicate the existence of contingent fee clauses in IPO project business agreements with accounting firms, where some fees are collected after the fundraising is completed or upon registration and review completion, making some fees dependent on the final outcome of the IPO project. This suggests that the independence of accounting firms in China’s IPO market faces challenges. Indeed, research indicates that in China, the choice of auditors is more likely based on mutual practical needs rather than auditors’ capabilities (Xia et al., 2023) [42]. 

Fourth, in the process of IPO in China, the role of underwriters is crucial. They possess a certain degree of discretionary power but also have a significant influence on the selection of accounting firms. The literature extensively documents the key role of underwriters in facilitating companies to go public (Li et al., 2021) [43]. Within China’s unique market regulatory environment, the status of underwriters is further highlighted. Since 2005, the China Securities Regulatory Commission has adopted a pure book-building system, further strengthening the role of underwriters. Underwriters serve not only as the primary intermediary agency in IPO projects but also wield certain discretionary powers. As mentioned earlier, under the stock approval system, especially when regulatory bodies impose a cap on issuance prices and provide window guidance, the core work of underwriters includes not only sales and pricing but also pre-listing counseling, in collaboration with accounting firms, subject to regulatory review. In this complex process, the pricing of underwriting and auditing fees during the IPO is relatively flexible, resulting in uneven distribution, which is the outcome of a dynamic game among interested parties. According to statistics, since 2016, the average cost of A-share IPOs has been 50 million RMB, about 50% of the average value in the United States. Structurally, underwriting sponsorship fees account for 75% of the total costs, with a fee rate roughly consistent with the United States at about 6%. However, the annual audit fees post-listing are significantly lower than in the United States, with the average for A-shares being 1.55 million RMB and the median being 0.85 million RMB, both about one-tenth of the US figures. This situation provides an important backdrop for focusing our research on how the relational networks between underwriters and accounting firms affect the distribution of benefits. Therefore, understanding the influence within the relational networks of underwriters and accounting firms in the Chinese context, and how underwriters shape the distribution of benefits, is the core issue of this study.

## 4. Research Hypotheses

In the laws and regulations related to IPO in China, there are no specific requirements for the selection of auditors, only clear stipulations on audit content and quality. In the new stock market, due to information asymmetry, the choice of auditors depends more on the requirements for the auditors’ capabilities and independence (Anderson et al., 1993) [44]. Large auditing firms have more resources and stronger motivation to enhance their ability to provide high-quality audits (DeAngelo, 1981) [30]. Meanwhile, industry expertise is another attribute of auditor capability. Expertise increases auditors’ ability to identify accounting irregularities, thereby ensuring the effectiveness and reliability of accounting information (DeFond and Zhang, 2014) [45].

However, researchers have gradually realized that the explanatory power of auditor capability is limited. For example, in reverse mergers, the choice of auditors does not depend on auditor capability but on the relative bargaining power between the acquiring company and the listed company. In fact, the choice of auditors might be the result of underwriter recommendations, with underwriters tending to seek familiar accounting firms. Long-term business relationships and the trust they engender reduce the cost of communicating with auditors (Anderson et al., 1993) [44]. It has been found that 71.3% of underwriters engage in new rounds of cooperation with accounting firms they have previously worked with.

In the Chinese market, underwriters’ decision-making power significantly influences the choice of auditors, effectively realizing their intentions (underwriter reputation, audit independence, and auditor selection). When underwriters have cooperated with multiple accounting firms, how do they choose an accounting firm? According to the interest predation hypothesis, underwriters will choose or recommend accounting firms that bring them more private benefits (El-Khatib et al., 2015) [11]. Existing literature finds that underwriting fees typically account for 7% of the total funds raised. However, underwriting fees in China are subject to significant fluctuations. According to interviews with underwriters in mainland China, underwriting fees are determined through negotiation between the company and the underwriter. With the company setting the issuance fee, the higher the underwriter’s relative bargaining power over the accounting firm, the more likely they are to seek private gains by selecting a specific audit institution, thus increasing the proportion of underwriting fees. 

Ultimately, how does the more central position of lead IPO underwriters in the investment banking–accounting firm network affect the distribution of interests between the two? To answer this question, consider a lead IPO underwriter that has established connections with many other accounting firms through multiple IPO participations, with SNA capturing its position in these relationship networks. Let every underwriter in the network also establish connections with multiple accounting firms through repeated prior interactions. In this context, we can envision two ways in which the position of lead IPO underwriters in their relationship network could affect the distribution of interests between the two. First, underwriters in the underwriter–accounting firm network are more likely to access key information such as corporate information and audit information from the network, enhancing underwriters’ bargaining power. Second, connections between stakeholders within the network reduce the level of information asymmetry among internal nodes, especially between central underwriters and other institutions within the network, but increase information asymmetry and barriers for external institutions. In other words, close cooperation between underwriters and accounting firms does not make companies more knowledgeable about accounting firms. As the intermediary institution and recommender of accounting firms dominating the IPO project, underwriters embedded in social networks exert the greatest influence in shaping the distribution of interests through acquiring and exchanging information, resources, and other social capital. Given the issuance fee set by the company, underwriters might choose specific accounting firms, thus turning the right of selection to their own benefit, thereby increasing the proportion of underwriting fees to audit fees. Therefore, we propose our first hypothesis:

**H1.**  *Underwriters with greater influence will significantly increase the proportion of underwriting and auditing fees.*

The influence wielded by underwriters in the network of relationships with accounting firms extends beyond just audit quality. While it is true that the level of their influence can impact audit quality through factors like auditor independence, auditor professionalism, and audit work, the primary focus of Hypothesis H1 is the significant increase in the proportion of underwriting and auditing fees associated with more influential underwriters.

In the realm of auditor independence, it is worth noting that the existence of a relationship network between underwriters and accounting firms can indeed reduce the independence of the latter. Repeated collaborations between underwriters and auditors often signify a relationship that transcends mere professionalism. Scholars have observed (Xie and Yan, 2013) [46] that signing auditors, in practice, may not exhibit substantial independence. Prolonged auditor–client relationships have the potential to undermine audit quality (Tepalagul and Lin, 2015) [47]. A lack of auditor independence might lead to an overreliance on client management, possible underestimation of risks, and consequently, a more lenient audit approach with inadequate or inappropriate procedures (Chi et al., 2009) [48].

Concerning auditor professionalism, greater influence from underwriters may prompt accounting firms to compromise on their professional standards for audits. Since underwriters occupy a prominent position in the IPO process, they might prefer accounting firms that are more aligned with their goals and responsive to their needs, as opposed to those renowned for their audit quality and professionalism.

When it comes to audit work, a stronger influence from underwriters can reinforce their interests, often leading to a reduction in audit fees. Lower audit fees might prompt accounting firms to adopt cost-cutting strategies, such as reducing auditor numbers or the depth of audit procedures. This can undermine the rigor and comprehensiveness of audits, making it challenging to identify potential financial irregularities. As a result, auditors may be more likely to conduct non-in-depth audits, ultimately compromising audit quality (Dye, 1993) [49].

**H2.**  *Higher underwriter influence leads to a decline in audit quality.*

The H1 addresses the economic implications in terms of fee structures, while the H2 explores broader audit quality impacts. While the hypotheses are related, they each contribute to an understanding of a different aspect of the IPO process: the economic impact and the audit quality impact.

## 5. Data and Methods

### 5.1. Data and Sample Selection

First, we filter out all Chinese A-share companies listed on the Shanghai and Shenzhen Stock Exchanges from 2014 to 2022 whose data are available in the China Stock Market and Accounting Research (CSMAR) database. The construction of the underwriter–accounting firm bipartite network and company-specific financial data are derived from the CSMAR database. Our initial sample selection is as follows: (1) Due to the unique nature of enterprises in the financial sector, we exclude them from our research sample; (2) To avoid the influence of abnormal company data on our research results, we eliminate companies whose stock codes begin with “ST”; (3) We exclude observations missing data. Ultimately, we obtained 1468 company-year observations. To mitigate the impact of outliers, all continuous variables were winsorized at the 1% upper and lower levels.

### 5.2. Methods

Information entropy, a concept originally introduced by Claude Shannon, provides a mathematical framework to measure the uncertainty or randomness in a given system. In the context of underwriter relationship networks, this metric can be adapted to quantify the centrality of individual underwriters.

To calculate the centrality of underwriters using information entropy, we first need to construct a network model where nodes represent underwriters and accounting firms, and edges represent their relationships or collaborations. The strength of these relationships can be weighted based on factors such as the frequency or value of their collaborations.

Once the network is established, we can compute the centrality of each underwriter by considering their connectivity within the network. A highly central underwriter will have a large number of strong connections, indicating their importance in the network.

Information entropy comes into play when we consider the distribution of these connections. If an underwriter has a highly uneven distribution of connections (i.e., they are strongly connected to only a few other underwriters), their centrality will be reflected in a lower entropy value. Conversely, if their connections are more evenly distributed, the entropy value will be higher.

In summary, the use of information entropy to quantify the centrality of underwriters in the network provides a valuable tool for understanding their influence and role in the IPO process. This metric not only helps to identify key players but also sheds light on how their position affects the distribution of benefits and audit outcomes.

## 6. Measures of Underwriter Influence and Distribution of Interests

### 6.1. Characteristics Representing Underwriter Influence

We hypothesize that the extent of collaboration between underwriters and accounting firms in their network during an IPO influences the pattern of interest distribution in the IPO. If an underwriter and an accounting firm have previously participated in the same IPO, we consider them to be connected. We describe the position of underwriters within the bipartite network of connections with accounting firms using various SNA measures, which we refer to as centrality measures.

To calculate these centrality measures, we need to construct an adjacency matrix X, which is an N × M matrix (where N is the number of underwriters in the network, and M is the number of accounting firms). If underwriter i and accounting firm j have jointly participated in k IPOs, then x_ij_ = k; if underwriter i and accounting firm j have not jointly participated in any IPOs, then x_ij_ = 0.

**Figure 1:**
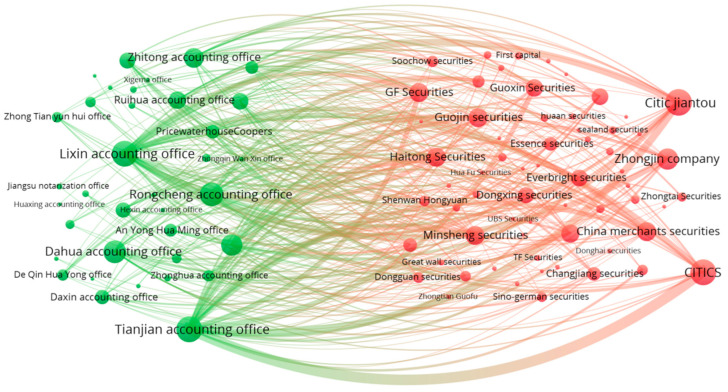
Underwriter–accounting firm network visualization.

Given the dynamic nature of the network of relationships between underwriters and accounting firms, we construct the underwriter centrality network on a rolling basis using data from the three years preceding the IPO. Specifically, the network of relationships for underwriters in 2021 is constructed based on every collaboration between underwriters and accounting firms from 2018 to 2020. A visualization analysis is presented in Figure 1:

In the constructed bipartite network of underwriters and accounting firms, network centrality is applied to measure the industry standing of underwriters. Given the more complex structure of bipartite networks compared to unipartite networks, this study uses three centrality measures for assessment in the bipartite network: Degree Centrality, Closeness Centrality, and Betweenness Centrality. The specific introductions to these measures are as follows:

Degree Centrality refers to the number of connections or degree of a node, which is the count of direct connections that the node has with other nodes. In the constructed bipartite network of underwriters and accounting firms, a higher degree centrality of an underwriter indicates a greater number of connected accounting firms, meaning the underwriter plays a more significant role in the entire network. The Formula (1) for calculating this measure is as follows:

(1) $${Degree}_{i}=\frac{\sum x_{ij}}{n-1}$$

where *x_ij_* Equation (1) represents that there is a cooperative relationship between underwriter *i* and *j*, and *n* equals the number of underwriters in the bipartite network of underwriters and accounting firms.

It measures how close a node is to all other nodes in the network. In the bipartite network of underwriters and accounting firms, closeness centrality can describe the role of a certain underwriter in transmitting information and resources among other accounting firms.

Specifically, in a bipartite network, underwriters and accounting firms represent two important types of nodes. If an underwriter has many connections with an accounting firm, then this underwriter has a high closeness centrality. This means they can more easily connect with other accounting firms and transmit information and resources to them; *d_ij_* is the shortest path length between underwriter *i* and accounting firm *j*. The Formula (2) for calculating this measure is as follows:

(2) $${Closeness}_{i}=\frac{\sum_{j=1}^{n}d_{ij}}{n-1}$$

In the bipartite network of underwriters and accounting firms, a higher betweenness centrality for an underwriter indicates that the underwriter is closer to other accounting firms, facilitating easier exchange of information and sharing of resources with other accounting firms, as well as enabling more efficient dissemination of information within the network; *b_jk_* is the number of shortest paths between accounting firm *j* and accounting firm *k*, while *b_jk_(i)* is the number of shortest paths between accounting firm *j* and accounting firm *k* that pass through underwriter *i*. The Formula (3) for calculating this measure is as follows:

(3) $${Betweenness}_{i}=\frac{2\sum_{j}^{n}\sum_{k}^{n}b_{jk}(i)}{n^{2}-3n+2}$$

Formula (4) for calculating the weighted average of degree centrality, closeness centrality, and betweenness centrality for each underwriter in the bipartite network of underwriters and accounting firms as the network centrality of the underwriter (LIU et al., 2021) [50] is as follows:

(4) $$Centrality=\frac{Degree+Closeness+Betweenness}{3}$$

### 6.2. Characteristics Representing Underwriter Interest Distribution

This paper adopts the interest distribution measure using the ratio of underwriting fees to auditing fees (SharingRatio) to represent the situation of underwriter interest distribution. Following the practices of existing literature, the model includes the following control variables: Big4 is a dummy variable, assigned a value of 1 if the company is audited by one of the Big Four accounting firms (PricewaterhouseCoopers (London, UK), Deloitte (London, UK), KPMG (Seoul, Republic of Korea), Ernst & Young (London, UK)), and 0 otherwise. Size is the natural logarithm of the company’s total annual assets, and age measures the company’s years since listing. REC is receivables, calculated as the latest annual receivables before listing divided by total assets, and INV is inventory, calculated as the latest annual inventory before listing divided by total assets. REC and INV reflect the complexity of the enterprise. Studies on the British audit market have found that the size and complexity of listed companies explain 79% of audit fees (Pronobis and Schaeuble, 2022) [51]. Lev is the leverage ratio, calculated as total liabilities at year-end divided by total assets at year-end. Roa is the return on assets, calculated by dividing net profit by the average balance of total assets. These two indicators comprehensively reflect the company’s performance and risk. Soe is a dummy variable, assigned a value of 1 for state-owned enterprises and 0 otherwise. Board reflects the number of members on the company’s board of directors. To test Hypotheses 1 and 2, this paper employs the following regression models:

(5) $$\mathrm{SharingRatio}=\alpha_{0}+\alpha_{1}centrality+\alpha_{j}\sum {control}_{i}+\varepsilon$$

(6) $$restate=\alpha_{0}+\alpha_{1}centrality+\alpha_{j}\sum {control}_{i}+\varepsilon$$

(7) $$cscore=\alpha_{0}+\alpha_{1}centrality+\alpha_{j}\sum {control}_{i}+\varepsilon$$

Each variable is defined as shown in Table 1:

## 7. Empirical Testing

Table 2 presents the descriptive statistics of the sample. The column N represents the number of samples, the column mean represents the mean of the sample, sd represents the standard deviation, min represents the minimum, max represents the maximum, and p50 represents the median. The average value of the SharingRatio is 8.1231, with a minimum value of 1.6308 and a maximum value of 46.5338, indicating that the ratio of underwriting fees to auditing fees for listed companies varies from 1.6308 to 46.5338. This suggests significant variation in this ratio among different companies, which could be influenced by various factors such as company size, business complexity, market environment, or pricing strategies between underwriters and auditing institutions. The average value of centrality is 0.3218, indicating that the centrality of nodes in the network is generally at a medium level. However, the large gap between its minimum value of 0 and maximum value of 0.4783 reveals clear centrality differences within the network. This means that the cooperative relationships between underwriters and accounting firms are not balanced, with some underwriters occupying more central positions in the network and having access to more resources.

Table 3 and Table 4 report the frequency of collaboration between lead underwriters and accounting firms and the statistics on the number of IPOs undertaken by lead underwriters, respectively. The results show that the average number of collaborations between lead underwriters and accounting firms is 8.57, indicating that lead underwriters and accounting firms have established relatively stable cooperative relationships in the IPO process. The maximum number of collaborations is 23, further highlighting the high degree of dependence between certain lead underwriters and specific accounting firms. A significant number of underwriters collaborate with accounting firms from between 2 to 10 times. The average number of IPOs undertaken by lead underwriters is 22.58 times, with a maximum of 123 times, demonstrating significant differences in the resources controlled by different underwriters in the IPO process. These differences are influenced by the underwriter’s market position, professional capability, capital strength, and the closeness of relationships with issuers, regulatory bodies, and other parties.

Table 5 reports the regression results on the impact of underwriter influence within the bipartite network of underwriters and accounting firms on the distribution of IPO interests. The regression controls for year and industry fixed effects, and the empirical results show a significant positive correlation between the centrality of underwriters in the bipartite network and the proportion of underwriting and auditing fees, significant at the 1% level. In other words, the higher the network centrality of the underwriter, indicating greater influence, the higher the proportion of underwriting and auditing fees. These regression results validate Hypothesis H1. In the competitive IPO market, higher network centrality of underwriters, indicating stronger influence, consolidates their interests, thereby leading to a decrease in the proportion of audit fees. 

Table 6 reports the regression analysis on the distribution of IPO interests within the bipartite network of underwriters and accounting firms, employing the degree centrality, closeness centrality, and betweenness centrality of underwriters as new explanatory variables to measure the level of underwriter influence. The regression controls for year and industry fixed effects and the results show that degree centrality, closeness centrality, and betweenness centrality have a significant positive correlation with the proportion of underwriting and auditing fees, reaching a significant level of 1%, thus validating the previous hypothesis.

Specifically, we find that in the bipartite network of underwriters and accounting firms, network centrality has an important impact on the distribution of IPO interests. The degree centrality of an underwriter reflects their level of connection with other accounting firms within the network, closeness centrality indicates the distance between the underwriter and other accounting firms, and betweenness centrality measures the importance of the underwriter as a mediator of information within the bipartite network. These centrality indicators, substituting for the core explanatory variable of underwriter influence–network centrality, allow for a more comprehensive consideration of the level of underwriter influence within the bipartite network.

In the main regression tests, we employ data from the three years prior to the IPO to construct the underwriters’ network centrality on a rolling basis. Since the network of relationships between underwriters and accounting firms is dynamically changing, here we construct the underwriters’ centrality network on a rolling basis using data from the five years preceding the IPO. Specifically, the network of relationships for underwriters in 2021 is based on every collaboration between underwriters and accounting firms from 2016 to 2020. Table 7 reports that in the five-year rolling bipartite network of underwriters and accounting firms, there is a significant positive correlation between underwriters’ network centrality and the proportion of underwriting and auditing fees, significant at the 1% level. This means that the higher the network centrality of the underwriter, indicating greater influence, the higher the proportion of underwriting and auditing fees, suggesting that the results are robust.

Table 8 reports the regression analysis conducted using different estimation methods. Column 1 controls for fixed effects of the year, industry, and region; Column 2 controls for individual and regional fixed effects; Column 3 controls for industry and regional fixed effects; Column 4 controls for the interaction of year and regional fixed effects; Column 5 controls for the interaction of year and industry as well as the interaction of year and regional fixed effects. The results still indicate that within the bipartite network of underwriters and accounting firms, there is a significant positive correlation between the centrality of underwriters in the network and the proportion of underwriting and auditing fees, significant at the 1% level.

To more comprehensively depict and understand the IPO market, we include market influence factors as control variables. We use the cumulative market return 120 trading days before the IPO, the standard deviation of the cumulative return, and the natural logarithm of the number of companies listed 120 trading days before the IPO plus one as standards for measuring market sentiment. The regression results in Table 9 show that after including the three control variables, there is a significant positive correlation between the centrality of underwriters in the bipartite network of underwriters and accounting firms and the proportion of underwriting and auditing fees, significant at the 1% level. The results remain robust.

In the bipartite network of underwriters and accounting firms, due to potential endogeneity between underwriters’ network centrality and other factors, this suggests that the centrality of underwriters in the network could be influenced by the underwriters’ reputation and the number of IPOs they handle. To mitigate the issue of endogeneity and accurately identify the independent impact of underwriters’ influence on the distribution of IPO interests, this paper selects the underwriters’ market share in IPOs as a proxy for the underwriters’ reputation and the number of IPOs handled by the underwriters as instrumental variables. An underwriter’s market share can indirectly reflect its reputation. In general, underwriters with larger market shares tend to have higher reputations because they are able to attract more IPO business. Therefore, it is reasonable to use market share as a proxy variable for reputation. The number of IPOs handled by the underwriters is a variable that is correlated with the centrality of the underwriter network, but not directly with the distribution of IPO benefits. It can be used as a valid instrumental variable to help determine the independent effect of underwriter influence on the distribution of IPO benefits. By using this instrumental variable, we can control for the number of IPOs handled by underwriters and thus more accurately estimate the impact of underwriter network centrality on other variables. The study employs a two-stage least squares (TSLS) regression analysis. The regression results show that Kleibergen–Paap rk LM statistic is significant at the 1% level, rejecting the hypothesis of insufficient identification of instrumental variables; the Cragg–Donald Wald F statistic is larger than the critical value at the 10% significance level of the Stock–Yogo weak instrumental variable identification F test which rejects the original hypothesis of weak instrumental variables; in conclusion, the instrumental variables selected in this paper are reasonable and reliable. The regression results in Table 10 show that the coefficients for the underwriters’ IPO market share, a measure of underwriters’ reputation, and the number of IPOs handled by underwriters are statistically significant and positive at the 1% level. This further validates the reliability of the conclusions, meaning that the underwriters’ IPO market share and the number of IPOs independently influence the distribution of IPO interests. The research findings address the endogeneity issue between underwriters’ network centrality and other factors and draw reliable conclusions about the impact of underwriters’ influence on the distribution of IPO interests.

Table 11 reports a regression analysis on abnormal underwriting fees within the bipartite network of underwriters and accounting firms. The regression results indicate a significant positive correlation between the centrality of underwriters and abnormal underwriting fees, reaching a significant level of 1%. 

Specifically, there is a positive correlation between the influence of underwriters and the underwriting fees they receive. Underwriters with higher influence are often able to raise funds more effectively for issuing companies, reduce issuance risks, and provide more value-added services. Consequently, issuing companies are more willing to pay higher fees for these services. Additionally, highly influential underwriters may also leverage their market influence to increase underwriting fees, thereby earning higher profits. This situation could lead to an abnormal increase in underwriting fees. For issuing companies and investors, understanding and reasonably evaluating this relationship is crucial for ensuring the efficient operation of the capital market and resource allocation.

Table 12 reports a regression analysis on the audit quality of listed companies underwritten within the bipartite network of underwriters and accounting firms. The audit quality of the companies is measured by the likelihood of financial statement restatements and the robustness of accounting practices using the Cscore model for the fiscal year of the company’s listing. The regression results show a decrease in audit quality for listed companies underwritten by underwriters with higher influence, specifically indicated by an increased probability of financial statement restatements during the year of listing and a reduction in financial robustness.

Specifically, audit quality is an important indicator of the accuracy and reliability of audit work. A decline in audit quality means there is an increased risk of significant errors or misleading information in the financial statements. This may compromise the independence and objectivity of the audit.

Further, this decline in audit quality is specifically reflected in an increased likelihood of financial statement restatements in the year of listing. Financial statement restatement refers to the process of identifying and correcting errors or misleading information in financial statements of previous years. If underwriters interfere with the preparation of financial statements during the listing process, these errors or misleading pieces of information are likely to be discovered and required to be restated in subsequent financial statements.

Lastly, such intervention and manipulation could also lead to weakened financial robustness of the company. Financial robustness refers to the ability of a company to maintain financial stability and continuous operation in the face of uncertainty and risk. When underwriters intervene in financial statements for their own benefit, they may conceal the true financial condition and risks of the company, thereby weakening its financial robustness.

Therefore, in the capital market, there should be strengthened regulation and constraint of underwriters to ensure the fairness and legality of their actions, protect the interests of investors, and promote the healthy development of the capital market.

Table 13 reports that in the heterogeneity test, a well-structured audit committee governance and the rationalization of market sentiment can mitigate the negative impact of underwriters’ influence to a certain extent. The regression results show a significant positive correlation between underwriters with higher influence and higher audit quality.

Specifically, as a crucial component of the corporate governance structure, the audit committee significantly influences the quality of financial reporting and information disclosure. The independence and expertise of the audit committee can enhance its supervisory capacity and governance effectiveness, improving the accuracy and reliability of financial reporting, thereby reducing the impact of underwriters’ influence on investors’ decisions.

The rationalization of market sentiment can also weaken the negative impact of underwriters’ influence. Underwriters often exploit market sentiment for their benefit, and the rationalization of market sentiment can reduce the occurrence of such manipulative behaviors, enabling investors to make more rational decisions, thus diminishing the negative impact of underwriters’ influence on the market.

In summary, a well-structured audit committee governance and the rationalization of market sentiment can mitigate the negative impact of underwriters’ influence to some extent, enhancing market stability and the quality of investors’ decisions.

## 8. Research Conclusions and Policy Recommendations

This paper introduces the concept of information entropy and selects 1468 IPOs of companies listed on the Shanghai and Shenzhen A-share markets from 2014 to 2022 as samples. Using the Ucinet (6.186) software, it constructs a bipartite network of underwriters and accounting firms during the IPO process and calculates the network centrality indicators within the bipartite network to measure the influence of underwriters. It empirically tests the significant role and mechanisms of underwriters’ influence on the distribution of IPO interests. Information entropy, as a tool to measure information uncertainty and complexity, provides a unique analytical perspective and methodological support for this study.

The main conclusions include the following points: when underwriters possess more significant influence, the ratio of underwriting fees to auditing fees significantly increases. Furthermore, more evidence suggests that higher influence often accompanies an increase in abnormal underwriting fees. Further research reveals a decline in audit quality for companies underwritten by underwriters with higher influence, specifically indicated by an increased likelihood of financial statement restatements in the year of listing and weakened financial robustness. Lastly, the study reveals that a well-structured audit committee governance and rationalization of market sentiment can mitigate the negative impact of underwriters’ influence to some extent. Information entropy, a measure of the uncertainty or randomness of information, plays a crucial role in this context. 

In financial markets, the cooperative relationship between underwriters and accounting firms may lead to moral hazards and conflicts of interest in some cases. Underwriters’ influence might also compromise auditors’ independence. There exists a close interest relationship between underwriters and clients, where underwriters can influence auditors’ interpretation of clients’ economic conditions and financial reports, potentially negatively impacting audit quality and investors’ interests. 

The research in this paper has the following limitations: Firstly, the scope of the study is relatively narrow, as this study mainly focuses on the underwriters’ influence in the IPO process and its impact on the distribution of benefits, without delving into the network of relationships among other financial institutions and how these networks jointly affect the entire process and outcome of IPOs. Future research could further expand the scope of the study to explore a wider range of relationship networks and mechanisms. Secondly, while the findings of this study provide empirical evidence of the important role and mechanisms of underwriters in the distribution of IPO benefits, more theoretical and empirical support is needed to further validate and refine these findings. Future research could explore underwriters’ influence and its long-term impact on the IPO market in depth from more perspectives and levels. Finally, we will not only focus on quantitative analysis in our follow-up study but will also include characteristics of underwriters and accounting firms for better qualitative analysis.

Based on our findings, we make a number of policy recommendations. Firstly, regulatory authorities must establish stricter rules and monitoring systems to oversee the relationship between underwriters and accounting firms, ensuring transparency and preventing conflicts of interest, while conducting regular audits to address any potential moral hazards. Secondly, to improve audit quality, accounting firms should be encouraged to maintain their independence from underwriters, with measures put in place to prohibit underwriters from influencing audit results. Thirdly, to enhance market competitiveness and transparency, regulators should consider policies that encourage diversity among underwriters and accounting firms, such as rotating underwriters and auditors, ultimately protecting investor interests and maintaining high-quality audits and underwriting services.

## Figures and Tables

**Table 1:** Definition of variables.

Stats	Definition
SharingRatio	The ratio of underwriting fees to auditing fees for listed companies
centrality	The average of degree centrality, closeness centrality, and betweenness centrality
restate	Restatement of financial statements in the year of a company’s listing
cscore	Cscore model
degree	Degree centrality: the number of accounting firms with which the underwriter collaborates
closeness	Closeness centrality: the underwriter’s ability to transmit information and resources
betweenness	Betweenness centrality: the underwriter’s capacity to act as a mediator
big4	A company audited by one of the Big Four (PricewaterhouseCoopers, Deloitte, KPMG, Ernst & Young) is marked as 1, otherwise 0
size	The natural logarithm of annual total assets
lev	Total liabilities at year-end divided by total assets at year-end
roa	Net profit divided by the average balance of total assets
soe	State-owned enterprises are marked as 1, others as 0
rec	The ratio of net receivables to total assets
inv	The ratio of net inventory to total assets
age	ln (year of listing—year of establishment +1)
board	The natural logarithm of the number of board members
market	Cumulative market return 120 trading days before the IPO
market.std	Standard deviation of daily market returns 120 trading days before the IPO
hot	Hot market, the number of companies listed 120 trading days before the IPO plus one, taken as the natural logarithm
underwriter	Underwriter market share
abnormal	Abnormal underwriting fees

**Table 2:** Descriptive statistics.

Stats	N	Mean	sd	Min	Max	p50
SharingRatio	1468	8.1231	6.7165	1.6308	46.5338	6.1038
centrality	1468	0.3218	0.0984	0.0000	0.4783	0.3397
degree	1468	0.2674	0.1405	0.0000	0.5590	0.2780
closeness	1468	0.6667	0.1536	0.0000	0.8080	0.7080
betweenness	1468	0.0313	0.0260	0.0000	0.1190	0.0270
big4	1468	0.0593	0.2362	0.0000	1.0000	0.0000
size	1468	20.8536	0.9203	19.3160	24.1817	20.6962
lev	1468	0.3516	0.1641	0.0653	0.7576	0.3361
roa	1468	0.0757	0.0366	0.0113	0.2165	0.0715
soe	1468	0.0899	0.2862	0.0000	1.0000	0.0000
rec	1468	0.1403	0.1026	0.0007	0.4537	0.1214
inv	1468	0.1130	0.0843	0.0004	0.4301	0.0961
age	1468	2.7494	0.3436	1.9459	3.5264	2.7726
board	1468	2.0852	0.1771	1.6094	2.4849	2.1972
market	1468	0.2189	0.3143	−0.2165	1.6135	0.1626
Market.std	1468	1.3983	0.4415	0.8573	3.3798	1.2764
hot	1468	5.0225	0.6278	3.0445	5.7268	5.2883

**Table 3:** Frequency of collaboration between lead underwriters and accounting firms.

Panel A						
Collaboration Frequency	Number of Underwriters	Mean	Standard Deviation	Minimum	Maximum	Median
1	7	1	0	1	1	1
2–5	20	3.55	1.01	2	5	4
6–10	17	7.4	1.46	6	10	7
11–15	9	12.4	1.13	11	14	12
16–20	8	18.8	0.5	17	20	18
21–25	4	22.5	0.58	22	23	22.5
Panel B						
Total	65	8.57	6.57	1	23	6

**Table 4:** Number of IPOs undertaken by lead underwriters.

Panel C						
Number of IPOs Undertaken	Number of Underwriters	Mean	Standard Deviation	Minimum	Maximum	Median
1–5	23	2.83	1.56	1	5	2
6–10	13	8.15	1.40	6	10	8
11–20	6	15.16	3.43	11	20	15
21–40	11	27.72	5.12	21	36	28
41–60	3	54.33	8.14	45	60	58
61–80	5	70.4	6.54	64	80	68
80 or more	4	96.5	18.86	83	123	90
Panel D						
Total	65	22.58	28.06	1	123	9

**Table 5:** Underwriter influence and interest distribution.

	(1)	(2)	(3)	(4)	(5)
Variables	Sharingratio	Sharingratio	Sharingratio	Sharingratio	Sharingratio
centrality	7.6713 ***	5.7153 ***	5.5301 ***	5.1673 ***	5.0994 ***
	(4.77)	(3.63)	(3.48)	(3.25)	(3.16)
big4		1.7443	1.7853	1.5122	1.4991
		(1.60)	(1.64)	(1.39)	(1.40)
size		1.6540 ***	1.4426 ***	1.4147 ***	1.3740 ***
		(5.47)	(4.55)	(4.47)	(4.43)
lev		−9.2104 ***	−8.8441 ***	−6.9868 ***	−7.0763 ***
		(−6.56)	(−6.34)	(−4.90)	(−4.99)
roa		5.7354	6.4035	4.6117	5.8556
		(1.10)	(1.22)	(0.88)	(1.11)
soe			2.1577 **	2.0407 **	1.9817 **
			(2.46)	(2.30)	(2.19)
rec				−4.8528 ***	−4.4990 ***
				(−2.96)	(−2.78)
inv				−6.7535 ***	−6.7457 ***
				(−3.12)	(−3.11)
age					−0.9517 *
					(−1.84)
board					1.5659
					(1.54)
Constant	6.7763 ***	−24.4491 ***	−20.1696 ***	−19.0026 ***	−19.1863 ***
	(3.96)	(−3.99)	(−3.13)	(−2.97)	(−2.72)
Observations	1468	1468	1468	1468	1468
Adjusted R-squared	0.033	0.093	0.100	0.108	0.110

Note: the value in brackets is t; *, ** and ***, respectively, indicate that the index is significant at the significance level of 10%, 5% and 1%.

**Table 6:** Substituting core explanatory variables.

	(1)	(2)	(3)	(4)	(5)	(6)
Variables	Sharingratio	Sharingratio	Sharingratio	Sharingratio	Sharingratio	Sharingratio
degree	6.1703 ***			4.3819 ***		
	(5.00)			(3.59)		
closeness		3.6677 ***			2.1966 **	
		(3.99)			(2.33)	
betweenness			25.5740 ***			17.1131 ***
			(3.86)			(2.63)
big4				1.4562	1.5426	1.5233
				(1.37)	(1.44)	(1.42)
size				1.3565 ***	1.4049 ***	1.3790 ***
				(4.37)	(4.52)	(4.42)
lev				−7.0323 ***	−7.1228 ***	−7.0436 ***
				(−4.96)	(−5.02)	(−4.95)
roa				5.8581	5.8821	6.0653
				(1.12)	(1.12)	(1.15)
soe				2.0097 **	1.9825 **	2.0283 **
				(2.23)	(2.19)	(2.24)
rec				−4.4658 ***	−4.5880 ***	−4.5539 ***
				(−2.76)	(−2.83)	(−2.81)
inv				−6.7373 ***	−6.8183 ***	−6.7155 ***
				(−3.12)	(−3.14)	(−3.12)
age				−0.9206 *	−0.9910 *	−0.9486 *
				(−1.78)	(−1.91)	(−1.83)
board				1.5875	1.5210	1.5955
				(1.56)	(1.50)	(1.57)
Constant	7.7261 ***	6.7094 ***	8.3198 ***	−18.3942 ***	−19.4972 ***	−18.3757 ***
	(4.62)	(3.92)	(5.14)	(−2.61)	(−2.75)	(−2.61)
Observations	1468	1468	1468	1468	1468	1468
Adjusted R-squared	0.037	0.028	0.031	0.113	0.108	0.109

Note: the value in brackets is t; *, ** and ***, respectively, indicate that the index is significant at the significance level of 10%, 5% and 1%.

**Table 7:** Using five-year rolling indicators.

	(1)	(2)	(3)	(4)
Variables	Sharingratio	Sharingratio	Sharingratio	Sharingratio
centrality_5	5.0505 ***			
	(2.83)			
degree_5		3.7661 ***		
		(3.27)		
closeness_5			2.3072 *	
			(1.96)	
betweenne_5				15.8649 **
				(2.08)
big4	1.5412	1.5064	1.5721	1.5611
	(1.44)	(1.41)	(1.47)	(1.45)
size	1.3797 ***	1.3664 ***	1.4085 ***	1.4027 ***
	(4.44)	(4.39)	(4.54)	(4.50)
lev	−7.0678 ***	−7.0161 ***	−7.1249 ***	−7.0789 ***
	(−4.99)	(−4.95)	(−5.02)	(−4.98)
roa	5.9691	5.9815	5.9705	6.0351
	(1.13)	(1.14)	(1.13)	(1.14)
soe	2.0158 **	2.0377 **	2.0050 **	2.0706 **
	(2.23)	(2.26)	(2.22)	(2.29)
rec	−4.4901 ***	−4.5501 ***	−4.5183 ***	−4.6975 ***
	(−2.77)	(−2.81)	(−2.79)	(−2.90)
inv	−6.8887 ***	−6.8398 ***	−6.9433 ***	−6.8425 ***
	(−3.17)	(−3.16)	(−3.19)	(−3.16)
age	−0.9476 *	−0.9265 *	−0.9844 *	−0.9764 *
	(−1.83)	(−1.79)	(−1.90)	(−1.88)
board	1.6256	1.6460	1.5555	1.6225
	(1.59)	(1.62)	(1.52)	(1.60)
Constant	−19.4019 ***	−18.5470 ***	−19.6860 ***	−18.9792 ***
	(−2.74)	(−2.63)	(−2.77)	(−2.69)
Observations	1468	1468	1468	1468
Adjusted R-squared	0.110	0.111	0.107	0.108

Note: the value in brackets is t; *, ** and ***, respectively, indicate that the index is significant at the significance level of 10%, 5% and 1%.

**Table 8:** Robustness test: changing estimation methods.

	(1)	(2)	(3)	(4)	(5)
Variables	Sharingratio3	Sharingratio3	Sharingratio3	Sharingratio3	Sharingratio3
centrality	5.4184 ***	5.4522 ***	5.4184 ***	5.4513 ***	4.5809 ***
	(3.39)	(3.09)	(3.04)	(3.60)	(3.08)
big4	1.5106	1.6012 **	1.5106 **	1.6155	1.4383
	(1.41)	(2.17)	(2.01)	(1.49)	(1.30)
size8	1.3769 ***	1.3692 ***	1.3769 ***	1.3914 ***	1.4952 ***
	(4.42)	(5.92)	(5.79)	(4.54)	(4.67)
lev	−6.9291 ***	−7.1888 ***	−6.9291 ***	−7.2057 ***	−6.8066 ***
	(−4.87)	(−5.29)	(−4.95)	(−5.05)	(−4.44)
roa1	6.4235	6.3597	6.4235	6.2317	5.6953
	(1.22)	(1.27)	(1.28)	(1.14)	(1.03)
soe	1.8594 **	1.9135 ***	1.8594 ***	1.8518 **	2.1743 **
	(2.07)	(3.07)	(2.84)	(2.25)	(2.44)
rec	−4.4445 ***	−4.5200 **	−4.4445 **	−4.7893 ***	−4.6626 ***
	(−2.74)	(−2.57)	(−2.44)	(−3.08)	(−2.71)
inv	−6.5207 ***	−5.2109 **	−6.5207 ***	−5.3808 ***	−7.8839 ***
	(−2.98)	(−2.54)	(−2.88)	(−2.70)	(−3.25)
firmage	−0.8686 *	−0.9435 *	−0.8686 *	−0.9404 *	−1.0192 **
	(−1.66)	(−1.91)	(−1.73)	(−1.87)	(−1.97)
board	1.3095	1.2445	1.3095	1.2077	1.5186
	(1.27)	(1.27)	(1.33)	(1.18)	(1.44)
Constant	−18.8054 ***	−18.4730 ***	−19.7054 ***	−18.1075 ***	−17.6367 **
	(−2.65)	(−3.72)	(−3.59)	(−2.62)	(−2.43)
Observations	1468	1468	1468	1468	1468
AdjustedR-squared	0.112	0.096	0.093	0.117	0.155

Note: the value in brackets is t; *, ** and ***, respectively, indicate that the index is significant at the significance level of 10%, 5% and 1%.

**Table 9:** Adding control variables, including market influence factors.

	(1)	(2)	(3)	(4)
Variables	Sharingratio	Sharingratio	Sharingratio	Sharingratio
centrality	4.7814 ***	4.9022 ***	4.8827 ***	4.8829 ***
	(2.99)	(3.06)	(3.05)	(3.05)
big4	1.2851	1.3391	1.3479	1.3477
	(1.21)	(1.27)	(1.28)	(1.28)
size	1.6304 ***	1.6176 ***	1.6167 ***	1.6166 ***
	(4.89)	(4.88)	(4.88)	(4.87)
lev	−7.0661 ***	−7.1597 ***	−7.1644 ***	−7.1647 ***
	(−4.99)	(−5.07)	(−5.07)	(−5.06)
roa	6.8090	6.9111	6.8566	6.8549
	(1.30)	(1.33)	(1.32)	(1.32)
soe	1.8997 **	1.8780 **	1.8844 **	1.8842 **
	(2.10)	(2.07)	(2.08)	(2.09)
rec	−5.0026 ***	−4.8909 ***	−4.8962 ***	−4.8969 ***
	(−3.07)	(−3.01)	(−3.01)	(−3.03)
inv	−6.7191 ***	−6.6149 ***	−6.5964 ***	−6.5959 ***
	(−3.10)	(−3.07)	(−3.06)	(−3.07)
age	−0.7889	−0.8206	−0.8250	−0.8250
	(−1.52)	(−1.58)	(−1.59)	(−1.59)
board	1.2875	1.2327	1.2273	1.2273
	(1.26)	(1.21)	(1.20)	(1.20)
market		1.9534 ***	1.8994 ***	1.9029 **
		(2.93)	(2.82)	(2.25)
Market.std			−0.2065	−0.2089
			(−0.35)	(−0.32)
hot				−0.0057
				(−0.01)
Constant	−24.6458 ***	−24.6324 ***	−24.2855 ***	−24.2615 ***
	(−3.27)	(−3.28)	(−3.25)	(−2.95)
Observations	1468	1468	1468	1468
Adjusted R-squared	0.119	0.122	0.121	0.121

Note: the value in brackets is t; ** and ***, respectively, indicate that the index is significant at the significance level of 5% and 1%.

**Table 10:** Endogeneity test.

	(1)	(2)	(3)	(4)
Variables	Centrality	Sharingratio	Centrality	Sharingratio
marketshares	1.7229 ***			
	(23.27)			
underwriter			0.0815 ***	
			(36.99)	
centrality		7.8174 **		6.7170 ***
		(2.46)		(2.73)
big4	−0.0099	1.4594	−0.0061	1.4754
	(−1.12)	(1.38)	(−0.94)	(1.40)
size	0.0003	1.3380 ***	0.0010	1.3526 ***
	(0.09)	(4.35)	(0.49)	(4.43)
lev	0.0067	−7.0437 ***	0.0008	−7.0569 ***
	(0.41)	(−5.01)	(0.06)	(−5.03)
roa	−0.0096	5.7697	0.0055	5.8045
	(−0.16)	(1.11)	(0.13)	(1.12)
soe	0.0029	1.9490 **	0.0096 *	1.9623 **
	(0.46)	(2.18)	(1.81)	(2.19)
rec	−0.0254	−4.3729 ***	−0.0282	−4.4240 ***
	(−1.12)	(−2.71)	(−1.52)	(−2.77)
inv	−0.0260	−6.6448 ***	−0.0160	−6.6856 ***
	(−0.87)	(−3.10)	(−0.70)	(−3.14)
age	0.0016	−0.9154 *	0.0031	−0.9301 *
	(0.29)	(−1.78)	(0.69)	(−1.82)
board	−0.0184	1.6139	−0.0065	1.5945
	(−1.46)	(1.59)	(−0.64)	(1.58)
Constant	0.2968 ***	−19.4853 ***	−0.0466	−19.3642 ***
	(4.67)	(−2.78)	(−0.92)	(−2.76)
Observations	1468	1468	1468	1468
Adjusted R-squared	0.360	0.109	0.591	0.110
Kleibergen–Paap rk LM statistic		420.963		344.661
Cragg–Donald Wald F statistic		608.368		1760.601
		[16.38]		[16.38]

Note: ***, **, and * indicate significance levels at 1%, 5%, and 10%, respectively; within [ ] are the critical values at the 10% level for the Stock–Yogo weak identification test. Same as below. Columns (2) and (4) report the regression results of the second stage of the instrumental variable method.

**Table 11:** Underwriter influence and abnormal underwriting fees.

	(1)	(2)	(3)	(4)
Variables	Abnormal	Abnormal	Abnormal	Abnormal
centrality	0.0213 ***			
	(3.08)			
degree		0.0099 **		
		(1.96)		
closeness			0.0175 ***	
			(4.13)	
betweenness				0.0210
				(0.79)
big4	0.0013	0.0013	0.0013	0.0015
	(0.45)	(0.47)	(0.48)	(0.54)
size	−0.0068 ***	−0.0067 ***	−0.0068 ***	−0.0066 ***
	(−7.63)	(−7.51)	(−7.67)	(−7.40)
lev	0.0025	0.0025	0.0024	0.0024
	(0.44)	(0.43)	(0.41)	(0.41)
roa	−0.0094	−0.0090	−0.0098	−0.0086
	(−0.50)	(−0.48)	(−0.52)	(−0.46)
soe	0.0069 **	0.0070 **	0.0066 **	0.0071 **
	(2.48)	(2.55)	(2.40)	(2.57)
rec	0.0360 ***	0.0356 ***	0.0362 ***	0.0353 ***
	(4.84)	(4.78)	(4.88)	(4.71)
inv	0.0110	0.0106	0.0111	0.0105
	(1.17)	(1.13)	(1.19)	(1.10)
age	0.0002	0.0001	0.0001	−0.0000
	(0.10)	(0.07)	(0.07)	(−0.00)
board	0.0121 ***	0.0119 ***	0.0120 ***	0.0118 ***
	(3.01)	(2.97)	(3.02)	(2.93)
Constant	0.1139 ***	0.1167 ***	0.1093 ***	0.1165 ***
	(5.17)	(5.31)	(4.95)	(5.31)
Observations	1468	1468	1468	1468
Adjusted R-squared	0.070	0.067	0.074	0.065

Note: the value in brackets is t; ** and ***, respectively, indicate that the index is significant at the significance level of 5% and 1%.

**Table 12:** Underwriter influence and audit quality.

	(1)	(2)	(3)	(4)	(5)	(6)	(7)	(8)
Variables	Restate	Restate	Restate	Restate	Cscore	Cscore	Cscore	Cscore
centrality	0.1704 **				−0.7114 **			
	(2.34)				(−2.17)			
degree		0.1013 *				−0.4119 **		
		(1.81)				(−1.97)		
closeness			0.1108 ***				−0.4834 **	
			(2.85)				(−1.96)	
betweenne				0.5235				−1.8197 *
				(1.50)				(−1.92)
big4	0.0206	0.0204	0.0215	0.0215	0.1424	0.1430	0.1388	0.1373
	(0.62)	(0.61)	(0.65)	(0.65)	(0.78)	(0.79)	(0.76)	(0.75)
size	−0.0030	−0.0027	−0.0026	−0.0027	0.1022 **	0.1007 **	0.1008 **	0.0994 **
	(−0.25)	(−0.22)	(−0.21)	(−0.22)	(2.29)	(2.27)	(2.26)	(2.22)
lev	0.0533	0.0537	0.0520	0.0541	−0.1366	−0.1380	−0.1313	−0.1381
	(0.75)	(0.75)	(0.73)	(0.76)	(−0.67)	(−0.67)	(−0.64)	(−0.67)
roa	−0.0438	−0.0421	−0.0452	−0.0369	0.4410	0.4334	0.4482	0.4134
	(−0.22)	(−0.21)	(−0.22)	(−0.18)	(0.48)	(0.47)	(0.48)	(0.44)
soe	0.0080	0.0092	0.0070	0.0096	−0.2942 **	−0.2996 **	−0.2894 **	−0.3011 **
	(0.29)	(0.34)	(0.26)	(0.35)	(−2.01)	(−2.05)	(−1.97)	(−2.06)
rec	0.2034 **	0.2017 **	0.2029 **	0.2010 **	0.1818	0.1894	0.1823	0.1954
	(2.20)	(2.18)	(2.19)	(2.17)	(0.76)	(0.79)	(0.75)	(0.81)
inv	−0.1580	−0.1597	−0.1584	−0.1576	0.3867	0.3946	0.3875	0.3898
	(−1.52)	(−1.54)	(−1.53)	(−1.51)	(1.29)	(1.31)	(1.29)	(1.30)
age	−0.0100	−0.0100	−0.0108	−0.0101	0.0677	0.0679	0.0708	0.0696
	(−0.44)	(−0.43)	(−0.47)	(−0.44)	(0.92)	(0.93)	(0.95)	(0.95)
board	−0.0529	−0.0533	−0.0536	−0.0523	−0.0402	−0.0381	−0.0376	−0.0404
	(−1.16)	(−1.17)	(−1.18)	(−1.15)	(−0.26)	(−0.25)	(−0.24)	(−0.26)
Constant	0.4605 *	0.4846 *	0.4353	0.4869 *	−3.2240 ***	−3.3240 ***	−3.1104 ***	−3.3288 ***
	(1.68)	(1.76)	(1.58)	(1.77)	(−2.91)	(−2.99)	(−2.80)	(−2.99)
Observations	1468	1468	1468	1468	1468	1468	1468	1468
Adjusted R-squared	0.106	0.105	0.106	0.105	0.124	0.123	0.125	0.123

Note: the value in brackets is t; *, ** and ***, respectively, indicate that the index is significant at the significance level of 10%, 5% and 1%.

**Table 13:** Corporate governance, auditor governance, and market sentiment heterogeneity test.

	(1)	(2)	(3)	(4)
Variables	Sharingratio	Sharingratio	Sharingratio	Sharingratio
	Low Governance			High Sentiment
centrality	6.2903 ***	2.9771	1.9797	9.0129 ***
	(2.76)	(1.34)	(0.98)	(3.35)
big4	2.0610	0.8364	3.0200 **	0.0207
	(1.25)	(0.54)	(2.05)	(0.01)
size	1.3649 ***	1.4405 ***	1.0075 **	1.8214 ***
	(3.16)	(3.19)	(2.24)	(4.49)
lev	−7.8217 ***	−6.3330 ***	−6.0594 ***	−7.8383 ***
	(−3.83)	(−3.09)	(−3.25)	(−3.51)
roa	0.5830	13.1172	12.5584 *	1.2893
	(0.09)	(1.51)	(1.71)	(0.17)
soe	2.2721 *	2.2491 *	2.7342 **	1.1336
	(1.72)	(1.92)	(2.06)	(0.98)
rec	−4.2518 **	−4.1362 *	−2.6905	−6.7537 ***
	(−1.99)	(−1.71)	(−1.15)	(−2.91)
inv	−5.7637 *	−7.7964 ***	−1.2259	−11.8110 ***
	(−1.82)	(−2.65)	(−0.43)	(−3.44)
age	−0.9434	−1.1424	−1.2121 *	−0.6761
	(−1.43)	(−1.48)	(−1.68)	(−0.90)
board	0.9636	2.0614	1.6172	1.6582
	(0.73)	(1.34)	(0.98)	(1.31)
Constant	−19.1325 *	−18.2867 *	−13.5615	−25.8886 ***
	(−1.87)	(−1.86)	(−1.29)	(−2.99)
Observations	734	734	734	734
Adjusted R-squared	0.124	0.087	0.074	0.144

Note: the value in brackets is t; *, ** and ***, respectively, indicate that the index is significant at the significance level of 10%, 5% and 1%.

## Data Availability

All data used are available in published sources.

## Appendix A

**Table A1:** Summary of issuance expenses for newly listed companies from 2011 to 2022.

Summary of Issuance Expenses for Newly Listed Companies from 2011 to 2022										
Metric	Total Fundraising Amount (Billion)	Issuance Expenses (Million)	Underwriting Fees (Million)	Audit Fees (Million)	Legal Fees (Million)	Disclosure Fees (Million)	Other Fees (Million)	Issuance Expenses as % of Total Fundraising	Audit Fees as % of Total Fundraising	Ratio of Issuance Fees to Underwriting Fees
Average	10.16	6719.56	5138.48	643.47	372.56	371.21	252.74	10.39%	1.14%	1.37
Median	5.56	5502.53	3950	552.85	300	424.53	80	9.77%	0.88%	1.34
Maximum	532.30	62,432.69	60,173.26	7016.46	3750	845.48	8421.38	34.88%	6.92%	2.39
Minimum	0.38	898.82	400	35	5.3	28.3	2.67	0.13%	0.12%	1.03
Standard Deviation	24.61	4565.44	4139.06	501.57	286.18	173.64	465.63	0.0455	0.0102	0.1865
